# Supplementary material for: Using machine learning to determine the shared and unique risk factors for marijuana use among child-welfare versus community adolescents
Source: PLoS One. 2022 Sep 21;17(9):e0274998. doi: 10.1371/journal.pone.0274998 (PMC9491564; doi:10.1371/journal.pone.0274998)
Supplement: S1 Table — (DOCX) [file pone.0274998.s001.docx]

**Supporting information**

| **S1 Table. Performance metrics for the three machine learning approaches for non-imputed (raw) data** | | | | | | | |
| --- | --- | --- | --- | --- | --- | --- | --- |
|  | **CW** | | |  | **Non-CW** | | |
|  | AUC | Precision | Recall |  | AUC | Precision | Recall |
| Logistic Regression | 0.82 | 0.75 | 0.75 |  | 0.85 | 0.71 | 0.66 |
| Lasso | 0.81 | 0.71 | 0.72 |  | 0.85 | 0.71 | 0.66 |
| SVM | 0.82 | 0.76 | 0.76 |  | 0.83 | 0.73 | 0.70 |
