# Supplementary material for: Using machine learning to determine the shared and unique risk factors for marijuana use among child-welfare versus community adolescents
Source: PLoS One. 2022 Sep 21;17(9):e0274998. doi: 10.1371/journal.pone.0274998 (PMC9491564; doi:10.1371/journal.pone.0274998)
Supplement: Attachment — Submitted filename: PLoS One Review_PONE-D-21-15650.docx [file pone.0274998.s002.docx]

PLoS One Review:

Using machine learning to determine the shared and unique risk factors for marijuana use among child-welfare versus community adolescents

1. I suggest more extension and detail in how decision trees work in ML techniques. No reference in lines 56-58: “Not only can ML provide potentially more accurate predictive models, but techniques such as decision trees can provide new insights into non-linear relationships.”.
2. Lines 82-83: “there are no studies delineating *both the shared and unique risks*” is the italic necessary?
3. Why did CW=child welfare and non-CW turn into Line 117: maltreated and comparison groups? Please have uniformity.
4. What does a “biracial” participant’s race mean?
5. According to data analysis, why do not present results before and after multiple imputations? The missingness proportion is less than 2%, but if you apply multiple imputations, results sure present how data and results look before imputation.
6. Here is one serious concern with the analytical approach: Lines 242-243 describe how “generally a value higher than 0.7 designates a good model, and higher than 0.8 a strong model”. Table 3 presents AUC for all models between 0.79 and 0.83. That means all models (i.e., Logistic Regression, Lasso and SVM) are good or strong. Why do you opt for different models under small and marginal differences?
7. Following the previous comment in Line 237- 239: In linear SVM, one can interpret the magnitude and sign of the coefficient in the linear hyperplane similarly to the coefficients in Logistic Regression and Lasso. What references do you have for this affirmation? Also, it is unclear whether your study is a comparison (e.g., CW=child welfare and non-CW), but you might use non-comparable models (e.g., Logistic Regression vs SVM). Unusually, two different models are recognized for two independent samples under the same analysis. For CW, it is SVM, and for N-CW, it is the Logistic Regression. It is necessary to have a better explanation and justify with technical background why a model may be more applicable to one group and not to the other. This point needs a deep rationale for your data analysis proposal.
8. Line 272 It is “perturbed” or “permuted”?
9. Why do you use the restrictions of the Lasso model and the technique called Backward Feature Selection? It seems like there are too many restrictions for the exploration of Line 46: “further work to delineate the relative importance of known predictors of substance use /…/”.
10. How sensitive is the Permutation Feature Importance (PFI) to the SVM and the logistic regression results?
11. It is not easy to interpret the values from the Permutation Feature Importance (PFI) analysis. However, if the arbitrary selection of models we asked about in point 7 is justifiable, at least all results in all models should be presented for a fair comparison.
12. Since the authors claim that this is a study “Line 396-398: to provide evidence regarding the comparability of risk factors for substance use among CW-involved versus non-CW-involved youth, but the first to so within one study and using machine learning approaches.”, it is capital to clarify the analytic concerns mentioned before. The richness of their approach will reside in how well the points 5 to 11 are corrected and rationalized.
